# Redox proteins of hydroxylating bacterial dioxygenases establish a regulatory cascade that prevents gratuitous induction of tetralin biodegradation genes

**DOI:** 10.1038/srep23848

**Published:** 2016-03-31

**Authors:** Laura Ledesma-García, Ana Sánchez-Azqueta, Milagros Medina, Francisca Reyes-Ramírez, Eduardo Santero

**Affiliations:** 1 Centro Andaluz de Biología del Desarrollo, Universidad Pablo de Olavide/Consejo Superior de Investigaciones Científicas/Junta de Andalucía, and Departamento de Biología Molecular e Ingeniería Bioquímica, Seville, Spain; 2 Departamento de Bioquímica y Biología Molecular y Celular, and Instituto de Biocomputación y Física de Sistemas Complejos (BIFI), Universidad de Zaragoza, Zaragoza, Spain

## Abstract

Bacterial dioxygenase systems are multicomponent enzymes that catalyze the initial degradation of many environmentally hazardous compounds. In *Sphingopyxis granuli* strain TFA tetralin dioxygenase hydroxylates tetralin, an organic contaminant. It consists of a ferredoxin reductase (ThnA4), a ferredoxin (ThnA3) and a oxygenase (ThnA1/ThnA2), forming a NAD(P)H–ThnA4–ThnA3–ThnA1/ThnA2 electron transport chain. ThnA3 has also a regulatory function since it prevents expression of tetralin degradation genes (*thn*) in the presence of non-metabolizable substrates of the catabolic pathway. This role is of physiological relevance since avoids gratuitous and wasteful production of catabolic enzymes. Our hypothesis for *thn* regulation implies that ThnA3 exerts its action by diverting electrons towards the regulator ThnY, an iron-sulfur flavoprotein that together with the transcriptional activator ThnR is necessary for *thn* gene expression. Here we analyze electron transfer among ThnA4, ThnA3 and ThnY by using stopped-flow spectrophotometry and determination of midpoint reduction potentials. Our results indicate that when accumulated in its reduced form ThnA3 is able to fully reduce ThnY. In addition, we have reproduced *in vitro* the regulatory circuit in the proposed physiological direction, NAD(P)H–ThnA4–ThnA3–ThnY. ThnA3 represents an unprecedented way of communication between a catabolic pathway and its regulatory system to prevent gratuitous induction.

A wide range of aromatic compounds are major environmental pollutants, continuously discharged into the environment through industrial and urban activities causing irreversible damage to the biosphere. Microbial catabolic potential allows using a variety of hazardous compounds as growth substrates. Aromatic catabolic pathways usually initiate biodegradation by incorporation of oxygen into the aromatics rings catalyzed by Rieske non-haem iron diooxygenases, a reaction requiring oxygen and reducing equivalents from NAD(P)H. These multicomponent enzymes consist of a reductase, an oxygenase and, in some cases, an additional ferredoxin that mediates electron transfer between the former two components^1^. Further metabolism is achieved through hydroxylated aromatic intermediates. An efficient catabolic process imposes its catabolic genes being expressed at adequate levels only when the right substrates, those that the catabolic pathway can metabolize, are present, thus avoiding energetically wasteful production of catabolic enzymes and potentially wasteful consumption of NAD(P)H. Regulators of catabolic pathways are quite promiscuous, responding to distinct sets of structural analogues substrates and even to non-aromatic pollutants in some aromatic pathways^2^. This may result in a detrimental gratuitous induction of the pathway.

*Sphingopyxis granuli* strain TFA is able to grow with tetralin as a sole source of carbon and energy. Tetralin (1,2,3,4-tetrahydronaphthalene) is a bicyclic molecule composed of an aromatic and an alicyclic moiety, which is found at low concentrations in different crude oils, and it is also industrially produced for its use as an organic solvent. The degradation pathway has been characterized both at the biochemical and genetic levels^3^,^4^,^5^,^6^,^7^. As observed for other aromatic compounds, degradation of tetralin is initiated by dioxygenation of the aromatic ring, which is catalyzed by the tetralin dioxygenase enzymatic complex. This complex consist of a NAD(P)H-dependent ferredoxin reductase ThnA4 (NCBI protein accession number AAN26446), a ferredoxin ThnA3 (AAD52963), and a Rieske-type dioxygenase ThnA1/ThnA2 (AAN26443; AAN26444) that hydroxylates tetralin, forming the NAD(P)H-ThnA4–ThnA3–ThnA1/ThnA2 electron transport chain.

Expression of the tetralin biodegradation genes (*thn*) in *S. granuli* requires expression of the *thnR* and *thnY* regulatory genes. ThnR (AAU12855) is a LysR-type transcriptional activator that activates *thn* gene transcription in response to tetralin by binding to sites present at each of the four *thn* promoter regions^7^,^8^. ThnY (AAU12856) contains FAD and a plant-type [2Fe-2S] cluster and shows spectral features of the bacterial oxygenase-coupled NAD(P)H-dependent ferredoxin reductases. However, unlike ferredoxin reductases, purified ThnY is not reduced by NAD(P)H and instead has been recruited by the regulatory module. Addition of ThnY_ox_ to electrophoretic mobility shift assays containing ThnR and a probe bearing the *thn* promoters indicated that ThnY directly promotes *thn* transcription activation by ThnR^9^. In addition, the ferredoxin ThnA3 adds an “extra” regulation by preventing expression of the *thn* genes when the inducer of the pathway is a poor substrate for the dioxygenase, avoiding gratuitous induction of the pathway. Thus, mutant strains lacking ThnA3 activate transcription of the catabolic pathway to high levels in response to compounds other than tetralin. On the other hand mutants lacking either the α (ThnA1) or β (ThnA2) subunits of the dioxygenase showed very low levels of *thn* expression even in the presence of tetralin. Based on these data, it has been proposed that ThnA3 exerts its regulatory function based on a redox sensory mechanism. Thus, under conditions in which the catabolic pathway cannot efficiently metabolize the inducer molecule (deficient electron flux to the dioxygenase), ThnA3 accumulates in its reduced form and prevents induction of tetralin gene expression^10^,^11^. We have found that some ThnY variants with amino acid substitutions in the cofactor binding site completely loose the discrimination capacity of the *thn* system^11^. These results suggest a unique regulatory model whereby ThnA3 signals are transmitted to the regulatory system via modification of the redox state of ThnY, forming a new regulatory NAD(P)H–ThnA4–ThnA3–ThnY electron transport chain.

By using stopped-flow spectrophotometry methods and determining midpoint reduction potentials of the implicated proteins, we demonstrate in this paper that the ferredoxin ThnA3 is the electron partner of ThnY. We have further analyzed the electron transfer processes between the NADP(H)-ThnA4_ox_, ThnA4_red_-ThnA3_ox_ and ThnA3_red_-ThnY_ox_ couples, and reconstituted the electron transfer chain in the physiological direction proposed, ThnA4–ThnA3–ThnY. To our knowledge, this is the only case known where catabolic enzymes commonly associated with the electron transport chain of dioxygenase systems are coupled to the regulatory proteins to adjust gene expression in response to the catabolic flux in the cell.

## Results

### Electron transfer from ferredoxin ThnA3_red_ to ThnY_ox_

Previous *in vivo* studies using mutated ThnY lead to the proposal of a model in which the interaction of ThnA3_red_ with ThnY negatively modulates ThnY activity, through the reduction of ThnY and its subsequent inactivation^11^. The *in vitro* demonstration of the functionality of the ThnA3-ThnY electron transfer process would strongly support the model for modulation of the regulatory system.

**Figure 1:**
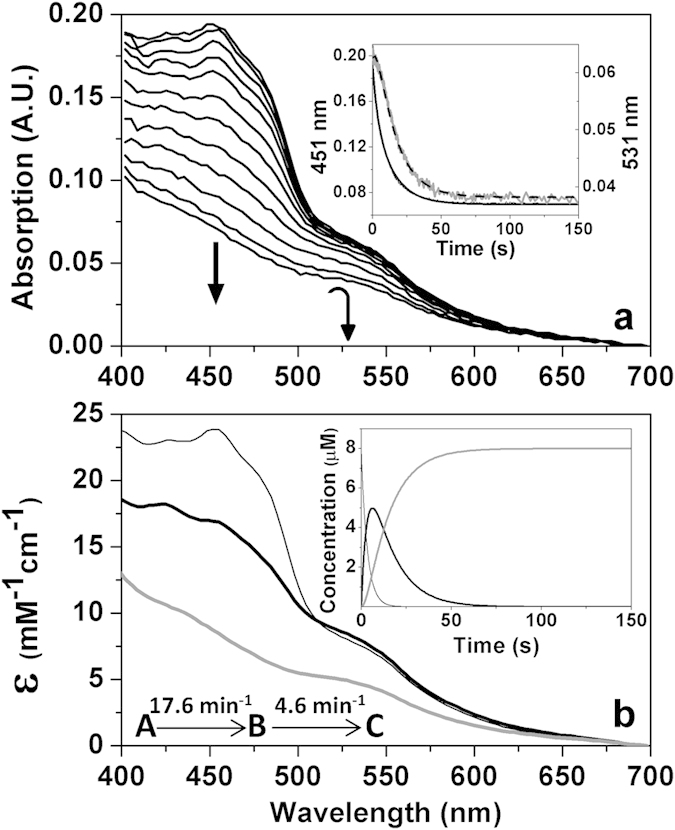
Anaerobic reduction of ThnY_ox_ by ThnA3_red_. (**a**) Spectral evolution of the reaction of ThnY_ox_ (~8 μM) with photoreduced ThnA3_red_ (~24 μM holoenzyme) as measured by stopped-flow spectrophotometry. Spectra recorded at 0.00128, 0.04736, 0.4109, 0.8614, 1.778, 3.488, 5.382, 8.25, 11.74, 17.8, and 54.38 s after mixing are shown. Direction of the spectral evolution is indicated by arrows. The inset shows the absorbance evolution at 451 nm (black line) and 531 nm (grey line) and their corresponding global fits to a two-steps model, A → B → C (bold black lines). (**b**) Spectroscopic properties of the intermediate pre-steady-state species. The inset shows the evolution of the obtained spectral species over the time. Species A, B and C are shown as continuous black thin, black bold and grey bold lines, respectively. Measurements carried out in potassium phosphate 50 mM, pH 7.4, NaCl 10 mM, glycerine 5%.

To investigate these facts, the hexahistidine-tagged versions of each of these proteins were purified by using metal chelate affinity and size exclusion chromatography. The His_6_-ThnY holoenzyme, containing FAD and a plant-type [2Fe-2S] cluster, was purified as previously reported^9^. ThnA3-His_6_ was here purified for the first time as a protein with an apparent molecular mass of 14 kDa, calculated from its mobility in SDS-PAGE, which agrees with the calculated from its coding sequence. The ThnA3 sequence bears the highly conserved metal-binding motif, CXHX_15–17_CX_2_H, containing the two cysteines and two histidines that co-ordinate the Rieske-type [2Fe-2S] cluster. Solutions of purified ThnA3_ox_ were brown-coloured and have the typical absorption spectrum of a Rieske-type [2Fe-2S] cluster with maxima at 280, 320, and 461 nm, and a shoulder at 580 nm, and became fully reduced by dithionite (Supplementary Fig. S1a). Hence, ThnA3 displays similar spectral properties to those of the related ferredoxins from benzene, toluene, biphenyl, carbazole and napthalene dioxygenase systems^12^. Nevertheless, cluster incorporation in recombinant ThnA3 was incomplete, with an average of 35% of [2Fe-2S] incorporation, an observation commonly associated with over-expressed iron-sulfur proteins^13^. Herein this percentage was considered to calculate the amount of holo-ThnA3 in our kinetic and potentiometric assays. The electron transfer process from ThnA3_red_ to ThnY_ox_ was analyzed by using stopped-flow spectroscopy, a method that can provide information on specific steps in the electron transfer reaction sequence and, therefore, will only imply ThnA3 in its holoprotein form when observing electron transfer. To do that we mixed a ~4-fold excess of an *in vitro* photoreduced solution of ThnA3 with ThnY_ox_, and evolution of the process was followed over the visible range observing a general decrease in the absorbance. As shown in Fig. 1a, the excess of ThnA3_red_ was able to fully reduce ThnY_ox_ under anaerobic conditions without detection of any traces of a semiquinone intermediate state. Different profiles for absorption evolution at 450 nm and 531 nm (inset Fig. 1a) as well as global analysis of the spectral evolution along the process were consistent with a two-step model, A → B → C, where three spectroscopic species can be distinguished (Fig. 1b). The initial species, A, practically results in the spectrum of ThnY_ox_. Conversion of A into B occurs with an observed rate constant, *k*_A→B,_ of 17.6 ± 1.5 min^−1^ under conditions of Fig. 1, with an absorbance decrease at the flavin band-I (around 450 nm) consistent with the two-electron reduction of the FAD cofactor of ThnY. In agreement, the spectrum of species B is consistent with the [2Fe-2S] cluster of ThnY remaining in the oxidized state. Spectroscopic changes for the final transformation of species B into C agree with the subsequent reduction of this [2Fe-2S] cluster (*k*_B→C_ = 4.6 ± 0.6 min^−1^). Thus, spectrum for species C is consistent with a fully reduced ThnY.

These results evidence that ThnA3 and ThnY are able to interact in solution and that ThnA3_red_ is able to directly transfer electrons to the regulatory protein ThnY. Moreover, our results indicate that this electron transfer follows an ordered mechanism, with FAD reduction of ThnY occurring first and being its iron sulfur-center the one reduced in the final step.

### Reduction of ferredoxin reductase ThnA4_ox_ by NAD(P)H

Ferredoxin reductases of the dioxygenases systems are known to supply reducing equivalents from NAD(P)H either directly to the terminal dioxygenase (Class I, two-component dioxygenase systems) or via an intermediary ferredoxin (Class II and III, three-component systems)^1^. *In vivo*, in the tetralin dioxygenase enzyme complex, (ThnA4–ThnA3–ThnA1/ThnA2), the ferredoxin reductase ThnA4 is postulated to transfer reducing equivalents from NAD(P)H to ferredoxin ThnA3^6^. ThnA4 encodes a 339-amino acid polypeptide, 45% identical to the ferredoxin reductases of the class III dioxygenase systems. These reductases share an N-terminal domain that contains a conserved Cys-*X*_4_-Cys-*X*_2_-Cys- *X*_29/30_-Cys motif that binds a plant-type [2Fe-2S] cluster, while the central and C-terminal domains contain the conserved motifs for flavin and NAD(P)H binding, respectively^9^. To *in vitro* analyze the reduction of ThnA3 under conditions closer to the physiological ones, (NAD(P)H–ThnA4–ThnA3), we also produced for first time recombinant ThnA4-His_6_ purified at homogeneity and studied its ability to accept electrons from pyridine nucleotides. Although ThnA4-His_6_ loses significant amounts of its flavin FAD cofactor during purification, upon FAD reconstitution its UV-visible air-oxidized spectrum revealed the typical absorption maxima at 276, 370, 421, and 461 nm, and a shoulder at about 550 nm, the later disappearing upon reduction by dithionite (Supplementary Fig. S1b). Thus, reconstituted ThnA4-His_6_ contains the expected FAD and [2Fe-2S] redox centers, showing similar spectral features to the reductases from the benzoate 1,2-dioxygenase^14^, napthalene dioxygenase^15^, 2-halobenzoate 1,2-dioxygenase^16^, and carbazole dioxygenase^17^ systems.

**Figure 2 f2:**
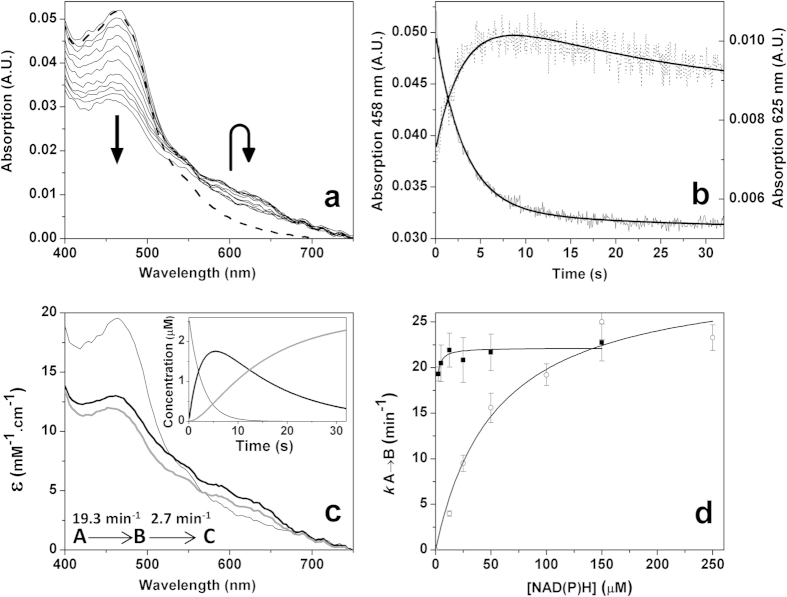
Reduction of ThnA4_ox_ by NADH. (**a**) Spectral evolution of ThnA4 (~2.5 μM) along reaction with NADH (~2.5 μM) as measured by stopped-flow spectroscopy under anaerobic conditions. The thick dashed black line corresponds to the spectrum of ThnA4_ox_ before mixing. Spectra recorded at 0.08064, 0.1626, 0.3264, 0.8179, 1.309, 2.456, 3.931, 5.242, 7.29, 10.07, and 30.23 s after mixing are shown. Directions of the spectral evolutions are indicated by arrows. (**b**) Evolution of the absorbance at 458 nm (line) and 625 nm (dotted line) and their corresponding global fits (bold lines) to a two-steps model, A → B → C. (**c**) Spectroscopic properties of the intermediate pre-steady-state species. The inset shows the evolution of the obtained spectral species over the time. Species A, B and C are shown as continuous black thin, black bold and grey bold lines, respectively. (**d**) Observed *k*_A→B_ values as a function of NADH (filled squares) and NADPH (open circles) concentrations. Lines represent data fit to Eq. 1. Experimental conditions as in Fig. 1.

Reduction of ThnA4_ox_ by NAD(P)H was also analyzed by fast-kinetic stopped-flow. Spectral evolution after mixing ThnA4_ox_ with NADH under anaerobic conditions clearly showed reduction of the enzyme (Fig. 2a). Similar results were also obtained under aerobic conditions and when NADPH was used as electron donor (not shown), indicating that ThnA4_ox_ can catalyze the oxidation of both coenzymes. Decrease in the absorbance at 461 nm upon reaction was concomitant with the appearance of a broad small long-wavelength band centered at 625 nm consistent with the appearance of a flavin-nicotinamide charge transfer complex along the reaction that finally bleaches (Fig. 2a[Fig f2]b). Global analysis of the spectral evolution shown in Fig. 2a was consistent with a two-step model (Fig. 2b,c). The first step, A → B, accounted for bleaching of the flavin band with the concomitant appearance of the long-wavelength charge transfer band. Transformation of B into C occurred with a rate constant ~7-fold slower than the initial process and accounted for the disappearance of the charge transfer complex band (Fig. 2a–c). When we used large coenzyme concentrations, and after a relatively long lag phase, conversion of C into a final D species was also observed, with spectral changes consistent with achieving full reduction of FAD and [2Fe-2S] clusters (not shown).

*k*_A→B_ values showed a saturation profile on NADH concentration that allowed us to estimate a limiting hydride transfer rate constant from NADH to ThnA4_ox_*, k*_red_^NADH^, of 22.1 ± 2.3 min^−1^, while suggesting a *K*_d_^NADH^ value lower than 0.4 μM. *k*_A→B_ values for NADPH showed a concentration saturation dependence that allowed fitting of the data to the equation describing binding at a single site followed by the hydride transfer processes and determination of the NADPH:ThnA4_ox_ dissociation constant (*K*_d_^NADPH^, 54 ± 13 μM) and the hydride transfer rate from NADPH to the FAD cofactor (*k*_red_^NADPH^ = 30.5 ± 2.1 min^−1^) (Fig. 2d). These parameters indicate that despite limiting rate constants for hydride transfer to ThnA4_ox_ are very similar for both coenzymes, the affinity of Thn4_ox_ for NADH is considerably higher than that for NADPH. These data are consistent with a higher efficiency for the process with NADH, indicating it as the preferred physiological hydride donor to ThnA4_ox_. Similar results were obtained regardless using aerobic or anaerobic conditions and the reverse reaction was undetectable under our experimental conditions, with the only exception of a very slow reverse reaction (*k*_reox_ = 0.16 min^−1^) for the process with NADPH under aerobic conditions. These results agree with observations in other related systems where the reductases, such as the ones of phthalate, toluene and benzene dioxygenases, are very specific for NADH^18^,^19^,^20^, while others less specific showed also preference for NADH over NADPH^15^.

### Reduction of ferredoxin ThnA3_ox_ by ferredoxin reductase ThnA4

**Figure 3:**
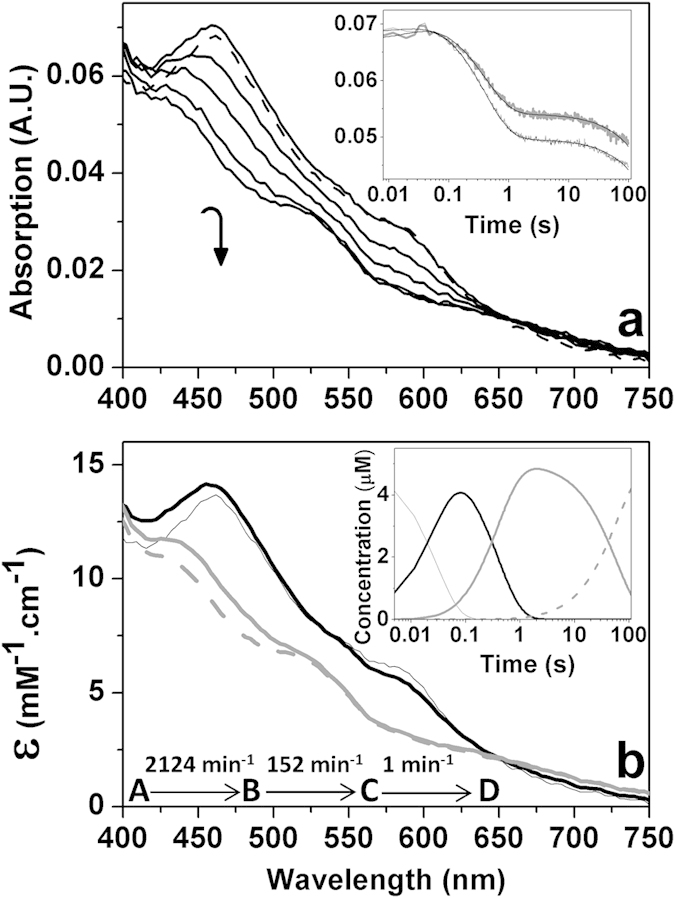
Anaerobic reduction of ThnA3_ox_ by ThnA4_red_. (**a**) Spectral evolution of the reaction of ThnA3_ox_ (~14 μM holoenzyme) with ThnA4_red_ (~5 μM, previously reduced with 50 μM NADH) as measured by stopped-flow spectroscopy under anaerobic conditions. Spectra recorded at 0.00384 (dashed line), 0.03968, 0.2138, 0.5082, 22.14, and 220.8 s after mixing are shown. The inset shows the evolution of the absorbance at 452 nm (grey bold line) and 462 nm (thin black line), as well as their corresponding global fits (bold lines) to a three-steps model, A → B → C → D. (**b**) Spectroscopic properties of the intermediate pre-steady-state species. The inset shows the evolution of the obtained spectral species over the time. Species A, B, C and D are shown as continuous black thin, black bold, grey bold and grey dashed lines, respectively. Experimental conditions as in Fig. 1.

Once demonstrated that ThnA4 is functionally reduced by NAD(P)H, we also analyzed its ability to transfer electrons from NADH to ThnA3_ox_. With this aim we followed the spectral evolution upon mixing under anaerobic conditions an excess of ThnA3_ox_ with ThnA4_red_, which was formed by previous incubation of ThnA4_ox_ with NADH (Fig. 3). The spectral shape of ThnA3_ox_ rapidly changed after mixing with ThnA4_red_ and its absorption peaks were displaced to 435 and 522 nm (Fig. 3a). These absorbance maxima are characteristic of reduced Rieske-type ferredoxins of aromatic systems such as BphA3 of the biphenyl dioxygenase complex from *Pseudomonas* sp. KKS102^21^, the ferredoxin_NAP_ component of naphthalene dioxygenase from *Pseudomonas*^17^ and the CarAc component of the carbazole 1,9α-dioxygenase^15^. The overall process fit to a three-step model (Fig. 3a, inset, and 3b). Species A resembled the ThnA3_ox_ spectrum. Transition of A into B was related with a slight increment in the absorption of the 460 band and a decrease in the 600 nm band, changes consistent with the production of the interaction between both proteins. Transition of B into C related with absorption decrease in the whole wavelength range consistent with reduction of ThnA3_ox_ by the NADH reduced ThnA4. Transformation of species C into D is a considerably slower process that accounts for a very small change in amplitude, probably related with final consumption of the excess of reduced coenzyme and the achievement of the steady-state equilibrium.

Altogether these results indicate that ThnA4 functions as a conventional NAD(P)H-dependent oxidoreductase of the dioxygenase systems, accepting a hydride from NAD(P)H by its flavin cofactor and transferring the two obtained electrons one at a time through its [2Fe-2S] cluster to the one-electron acceptor ThnA3 ferredoxin.

### Reconstitution of the regulatory electron transfer NADH–ThnA4–ThnA3–ThnY chain

**Figure 4:**
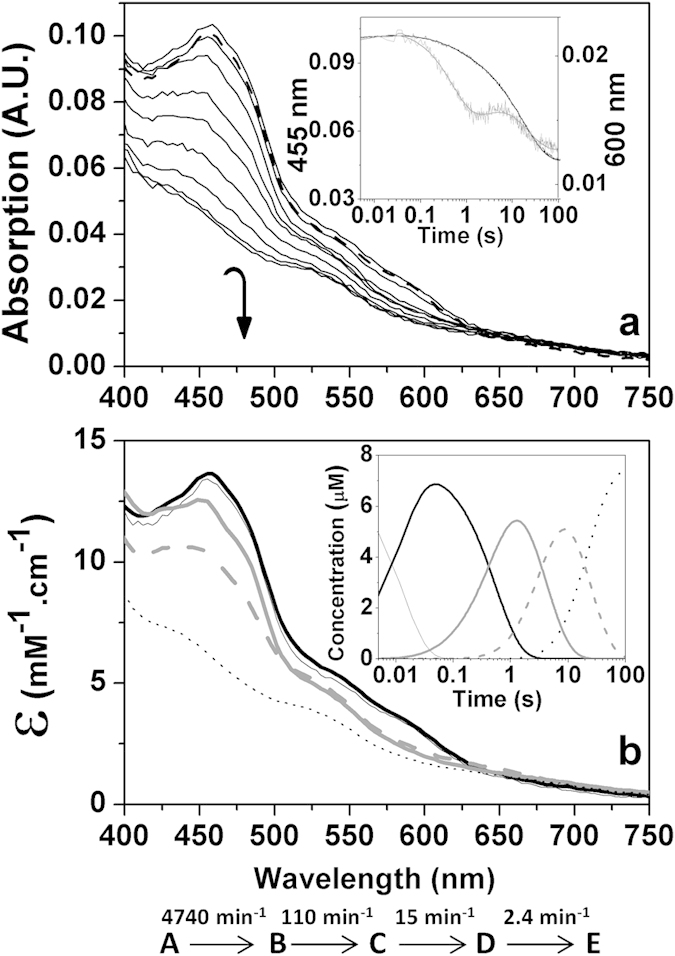
Reconstitution of the complete electron transfer chain: anaerobic reduction of ThnY_ox_ by NADH throughout ThnA3_ox_ and ThnA4_ox_. (**a**) Spectral evolution of the mixing ThnA4_red_ (~2.5 μM of protein preincubated with 50 μM NADH, in syringe 1) with a mixture containing ThnA3_ox_ (~25 μM holoenzyme) and ThnY_ox_ (~7.5 μM) (in syringe 2) in the stopped-flow equipment under anaerobic conditions. Spectra at 0.00384, 0.03968, 0.754, 0.887, 4.317, 8.982, 18.68, 38.31, 103, and 197 s after mixing are shown. The inset shows the spectral evolution at 455 nm (black line) and 600 nm (grey line), as well as and the corresponding global fits (bold lines) to a four-steps model, A → B → C → D → E. (**b**) Spectroscopic properties of the intermediate pre-steady-state species. The inset shows the evolution of the obtained spectral species over the time. Species A, B, C, D and E are shown as continuous black thin, black bold, grey bold, grey dashed and dotted lines, respectively. Experimental conditions as in Fig. 1.

Since ThnA3_red_ is able to reduce ThnY_ox_ when its iron cluster is photoreduced (Fig. 1) and NADH reduced ThnA4 (Fig. 2) is able to reduce ThnA3_ox_ (Fig. 3), we finally attempted to *in vitro* reconstitute the regulatory electron-transport chain in the proposed physiological direction by testing the reduction of the regulatory ThnY_ox_ by NADH *via* ThnA4 and ThnA3. Results for the anaerobic reconstitution of this system are shown in Fig. 4. Anaerobic stopped-flow measurements were carried out after mixing ThnA4_red,_ reduced by preincubation with NADH, with a mixture containing ThnA3_ox_ and ThnY_ox_. Spectral changes clearly indicated that ThnY_ox_ became completely reduced along the reaction time-course. Global analysis of the spectral evolution fit to a four-step model, A → B → C → D → E (Fig. 4a, inset and b). The initial intermediate species, A, is consistent with a mixture that mainly contains ThnA3_ox_, ThnY_ox_ and ThnA4_red_. Conversion of A into B shows a slight absorbance increase that can be related with complex formation among the proteins present in solution. Conversion of B into C is consistent with reduction of the [2Fe-2S] center of ThnA3 with a conversion rate constant, *k*_B→C_ = 110 min^−1^, very similar to that above obtained for the reduction of ThnA3_ox_ by ThnA4_red_ in the absence of ThnY (Fig. 3). Conversion of C into D indicates reduction of the FAD of ThnY, with an observed rate constant, *k*_C→D_ = 15 min^−1^, in the range described above by the first step in the analysis of the reduction of ThnY_ox_ by photoreduced ThnA3 (Fig. 1). Evolution to species E indicates final reduction of the three proteins with an observed rate constant of 2.4 min^−1^, again comparable with the values for the second steps of both the reduction of ThnY_ox_ by ThnA3_red_ (Fig. 1) and of ThnA3_ox_ by ThnA4_red_ (Fig. 3). Similar results were obtained under aerobic conditions, although in the presence of air the spectrum of ThnY_ox_ resulted regenerated at the end of the reaction. Control experiments in which ThnY_ox_ reduction was monitored in the absence of ThnA3, both under the same experimental conditions as well as in the presence of oxygen, showed that ThnA4_red_ was unable to reduce ThnY_ox_. These results confirm i) the requirement of ThnA3 for the efficient reduction of ThnY_ox_, as well as ii) the fact that we have reproduced *in vitro* the regulatory electron transfer chain from NADH to ThnY_ox_ via ThnA4 and ThnA3, as it is proposed to occur *in vivo*.

### Redox potentials values of ThnA4, ThnA3 and ThnY

**Figure 5:**
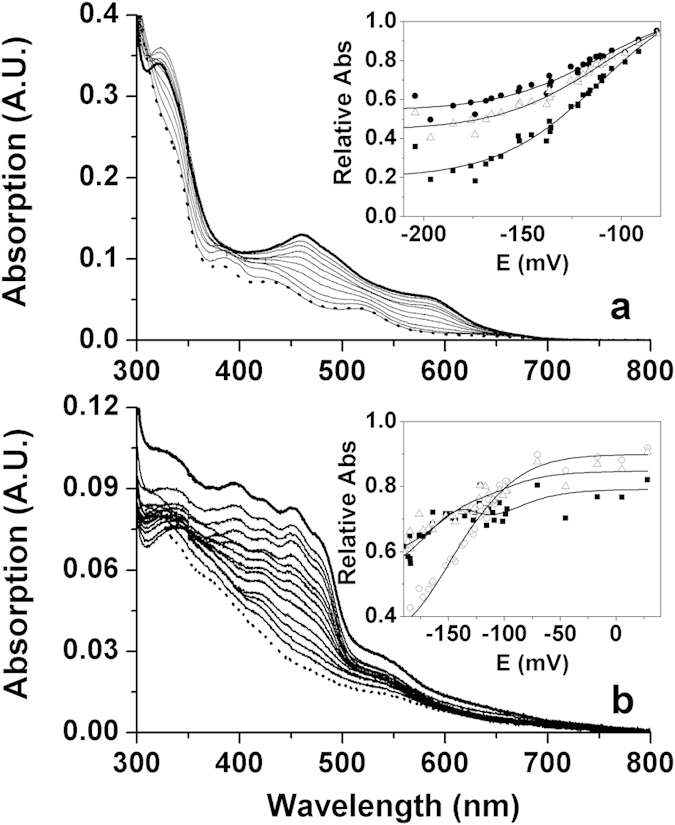
Potentiometric titrations of ThnA3 and ThnY. Spectral changes during photoreduction of (**a**) ThnA3 (∼20 μM) and (**b**) ThnY (∼5 μM). Buffers were supplied with 5-deazariboflavin, EDTA and the corresponding chemical mediators. Titrations were carried out at 15 °C in potassium phosphate 50 mM, pH 7.4 for ThnA3 and in 0.1 M HEPES, pH 7.4 for ThnY. Arrows indicate direction of spectral changes. The insets show multiple wavelength variation of the relative absorptions plotted against the redox potential of the solution (mV/SHE) at; (**a**) 590 (■), 520 (●) and 462 (Δ) nm for ThnA3 and (**b**) 530 (■), 450 (○) and 380 (Δ) nm for ThnY. Continuous lines show simultaneous fits of the different wavelength data to Eq. 2 for ThnA3 and Eq. 3 for ThnY.

Midpoint reduction potentials (*E*_m_), for ThnA3, ThnY and ThnA4, were determined by potentiometric titration and analysis of their visible spectra upon anaerobic photoreduction (Fig. 5). Spectral characteristics along photoreduction of ThnA3 were consistent with reduction of the [2Fe-2S] cluster in a process exchanging a single electron (Fig. 5a). Global fitting of data at several wavelengths to Eq. 2 allowed determination of a midpoint potential, *E*_ThnA3SFeox/red_, of −112 ± 5 mV (Fig. 5a inset). Spectra recorded upon photoreduction of ThnY showed the almost simultaneous reduction of its FAD (flavin band-I decrease at 454 nm) and [2Fe-2S] (absorption decrease at 530 nm) redox cofactors (Fig. 5b). Additionally, an absorption evolution at 380 nm consistent with the appearance of traces of anionic semiquinone along reduction was observed. Nevertheless, we observed that its maximal stabilization was too small to expect independent determination of the midpoint reduction potentials for the two individual oxido-reduction couples (*E*_ox/sq_ and *E*_sq/hq_). The plot of different wavelengths relative absorptions versus the measured potentials was globally fit to Eq. 3, allowing determination of *E*_ThnYFADox/hq_ and *E*_ThnYSFeox/red_ with values of −131 ± 8 mV and −136 ± 8 mV, respectively (Fig. 5b inset). Photoreduction of ThnA4 resulted in the extremely slow stabilization of the potential values after each reduction step, increasing the experimental time and the protein denaturation along the experiment. This enforced us to measure potentials and their corresponding absorption spectra at fixed times after each illumination event, that prevented accurate determination of midpoint potentials. Therefore, this experiment only suggested that *E*_ThnA4FADox/hq_ and *E*_ThnA4SFeox/red_ values must be in the −200 mV to −150 mV ranges respectively.

**Figure 6:**
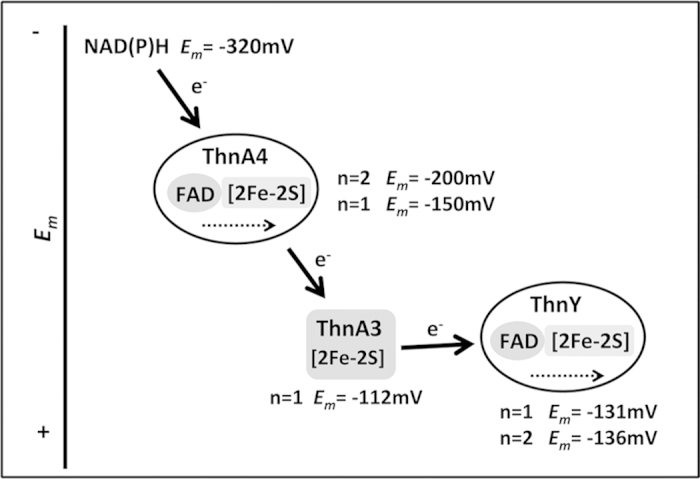
*In vivo* electron transfer pathway proposed for the reduction of ThnY by NAD(P)H via ThnA4-ThnA3. Midpoint reduction potentials are indicated for each redox cofactor. Complete results for their determination are shown in Fig. 5. Inter and intramolecular electron transfers are represented by arrows.

A schematic diagram with the midpoint reduction potentials and the inter and intra-molecular electron transfer steps is shown in Fig. 6. The ThnY midpoint reduction potential is slightly more electronegative than that of ThnA3, thus indicating that electron transfer in the direction ThnA3 → ThnY is only possible when ThnA3 accumulates in its reduced form (such condition will displace the actual reduction potential of ThnA3 to more negative values than the one determined as midpoint potential).

## Discussion

A feature of the regulatory systems of many biodegradation pathways is that the range of inducer molecules to which they respond is not the same as the range of substrates that the catabolic pathway can transform, therefore resulting in a superfluous and energetically wasteful production of catabolic enzymes unable to use the non-metabolizable molecules. Some regulators recognize as effectors molecules those with structural analogy to the substrate or even quite dissimilar compounds. Representative examples are DmpR and XylR, σ^54^-dependent regulators for catabolism of aromatics hydrocarbons such as (methyl)phenol and toluene/xylene respectively, which exhibit a very broad effector specificity^2^. Other catabolic pathways in order to prevent uncoordinated induction express the biodegradation genes not in response to the substrate but to some intermediate in the catabolism of the substrate^2^,^22^,^23^. However, this response implies high basal levels of expression to accumulate sufficient inducer intermediate to allow substantial degradation of the substrate. In addition, gratuitous induction is not fully prevented since some inducer intermediates may be produced through different peripheral routes that use different catabolic substrates.

**Figure 7:**
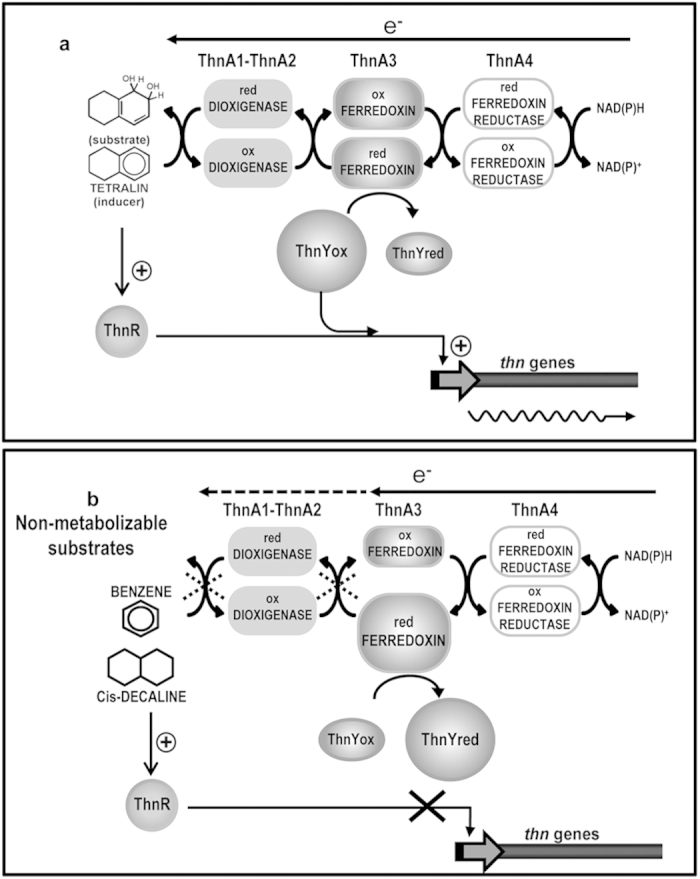
Model for the regulation of *thn* genes in response (a) to tetralin and (b) to non-metabolizable substrates. Blockage of electron transfer is represented by dotted crosses. The sizes of the circles indicate the relative abundance of that form of the protein according to the substrates supplied.

The *in vivo* model for *thn* gene regulation presented in Fig. 7 proposes that ThnA3 reports to the regulatory ThnR-ThnY system whether a potential inducer molecule is also a good substrate of the catabolic pathway, based in a redox sensory mechanism^10^. In recent years, much progress has been made in understanding how Fe-S clusters regulatory proteins reprogram the expression of genes in response to environmental stimuli. A challenging question is to relate both the *in vitro* reactions of Fe-S clusters with its physiological relevance^24^. Our model is quite unique, since ThnA3 is the only ferredoxin that takes part in oxidative hydroxylation of aromatic compounds known to be involved in regulation of gene expression. Therefore, it is a crucial question to elucidate the mechanism by which ThnA3 exerts its function.

The expression phenotypes of the ThnY mutants have provided genetic evidences indicating that control of the ThnY redox state is essential for efficient regulation of *thn* genes, since some of the *thnY* mutations in the electron cofactor binding sites alter the range of molecules able to activate the catabolic pathway. In this way, *thnY* mutant strains behave as the mutants lacking ThnA3, expressing *thn* genes in the presence of not suitable molecules such as *cis-*decalin, cyclohexane, *trans-*decalin, or benzene^11^. These findings and the *in vivo* model imply that under certain circumstances, electrons from NAD(P)H that are accumulated in ThnA3 are redirected towards ThnY instead of the dioxygenase, (NAD(P)H–ThnA4–ThnA3–ThnY electron chain), thus resulting in ThnY inactivation. To provide biochemical evidences of this regulatory electron transport chain, we have characterized the sequence for electron transport in this system. Our results clearly show that when the [2Fe-2S] cluster of ThnA3 is photoreduced or reduced by its physiological NADP(H) electron donor (ThnA4), it is able to reduce both the flavin and the [2Fe-2S] cluster of ThnY, thus strongly supporting the proposed regulatory model for the regulation of *thn*.

According to this model, ThnA3 is predominantly in its oxidized form in the presence of tetralin (Fig. 7a), the real substrate of the catabolic pathway. Reduction of ThnY by ThnA3 is minimal under these conditions, since electrons would be preferentially transferred to the dioxygenase, thus allowing ThnR and ThnY_ox_ to activate the *thn* promoters. In the absence of an efficient substrate that acts as an electron sink through the dioxygenation reaction (Fig. 7b), ThnA3 is accumulated in its reduced state. As a result, reduction of ThnY_ox_ by ThnA3_red_ will take place, switching ThnY into an abundant reduced form, thus impairing *thn* gene transcription.

Analogous proteins to ThnA4 and ThnA3 have been reported to function in the multienzyme systems that dioxygenate the aromatic substrates to cis-dihydrodiols. ThnA4 has several properties in common with the three-component oxygenase systems that catalyze reduction of ferredoxin from NAD(P)H: similar molecular weight, two prosthetic groups in a single polypeptide, a loosely bound molecule of FAD, and preference toward NADH. In fact the expected range for midpoint potentials for ThnA4 (*E*_ThnA4FADox/hq_ and *E*_ThnA4SFeox/red_) is in agreement with its role as NAD(P)H ferredoxin reductase. For comparison, in the phthalate dioxygenase reductase, PDR, the *E*_*m*_ for flavin is −230 mV, and the one-electron potential of [2Fe-2S] is −174^18^. Similarly ThnA3 shares common properties to the ferredoxin type protein of multicomponent dioxygenases enzymes, they are all one electron carriers, each has a Rieske-type [2Fe-2S], have similar molecular weights and they are rather specific for the dioxygenase system^1^. However, reduction potentials of the dioxygenase ferredoxins^25^ are approximately −150 mV. The *E*_ThnA3ox/red_ (−112 mV) is also comparable although clearly less negative than those found in ferredoxin Rieske counterparts. For comparison, the *E*_m_ for Rieske-type ferredoxins of aromatics benzene and biphenyl dioxygenases, or toluene 4-monooxygenase are −155, −157, and −173 mV, respectively^26^,^27^,^28^. On the other hand, the *E*_ThnYox/red_ (−131 and −136 mV) are less negative too than those of other ferredoxin reductases counterparts and slightly more negative than that of ThnA3 (Fig. 6). These values indicate that although when looking at midpoint reduction potentials electron transfer in the direction ThnA3 to ThnY is not favored, the situation can change when ThnA3 accumulates in the reduced form and its effective reduction potential becomes more negative. Moreover, these midpoint potentials fit well with the notion that ThnY_ox_ should not efficiently compete with the dioxygenase for the electrons coming from ThnA3 and, therefore, ThnY should only be reduced when ThnA3_red_ is highly accumulated *in vivo* and the equilibrium for the redox reaction changes. Actually, non-favored electron transfer from ThnA3 to ThnY may probably be essential for an efficient gene regulation because otherwise ThnY could always be reduced by ThnA3_red_, thus preventing transcription of *thn* genes under all conditions.

In conclusion, *in vivo* expression of the *thn* genes is prevented when reductive inactivation of ThnY occurs. This may happen (i) in the presence of an inducer molecule that is not a substrate of the dioxygenase and consequently high levels of ThnA3_red_ are accumulated^10^, (ii) in the *thnA1* or *thnA2* mutant strains, lacking of the dioxygenase subunits, in which all ThnA3_red_ is fully available to interact with ThnY and *thn* expression is impaired even in the presence of tetralin^10^ and (iii) when the inducer is also a substrate but its concentration is very low. Thus, under these conditions, redox equilibria favor electron transfer in the direction NADH  → ThnA4_FAD_ → ThnA4_[2Fe-2S]_ → ThnA3_[2Fe-2S]_ → ThnY_FAD_ → ThnY_[2Fe-2S]_ that we have reproduced *in vitro*. This way, the redox regulation exerted through ThnA3 not only prevents gratuitous induction by a non-metabolizable molecule but also may finely adjust the level of transcription of *thn* genes to the availability of the substrate.

## Methods

### Plasmids and strains constructions

Plasmids, strains, and primers used in this work are listed in Supplementary Table S1.

Plasmid pMPO785 to overproduce His_6_-ThnY was performed by PCR amplification of *thnY* with primers NdeI-thnY2 (5′AAAAACATATGGAAATCACCCTCATCC3′) and thnY-BamHI (5′AAAAAGGATCCTTACGAAACAGAAAAATGGTAAGG3′) using pMPO750 as template. *thnY* was cloned into pET-14b, PCR product and vector were cleavage with *Nde*I+*BamH*I.

The ThnA3-His_6_-overproducing plasmid pMPO760 was constructed amplifying *thnA3* by PCR with primers NdeI-thnA3 (5′CACATATGGGACGTAAGGTTAG3′) and thnA3-XhoI (5′GGCTCGAGATCAAGATCCGCGA3′), using pMPO751 as the template. The PCR product was cleaved with *Nde*I+*Xho*I and cloned into pET23a digested with the same enzymes.

Plasmid pMPO784, which contains *thnA4-His*_*6*_, was constructed by using primers NdeI-thnA4 (5′GACATATGGGCAGCGCGCGCAT3′) and thnA4-XhoI (5′GCCTCGAGGACAAAGCTGTC3′). The final DNA fragment was digested with *Nde*I+ *Xho*I, and was introduced into pET23b digested with the same enzymes.

*E. coli* DH5α was used as a host in all cloning procedures^40^. All DNA manipulations were performed according to standard procedures^29^. Plasmid DNA was transferred to competent cells of *E. coli* strains by heat shock transformation. When required, antibiotics were used at the following concentrations: ampicillin, 100 μg/ml, chloramphenicol, 15 μg/ml, and gentamicin, 10 μg/ml.

### Purification of recombinant proteins

Expression and purification of His_6_-ThnY (containing an N-terminal His_6_ tag), ThnA3-His_6_ (C-terminal His_6_ tag) and ThnA4-His_6_ (C-terminal His_6_ tag) proteins were performed as previously described^9^ with the following modifications: cultures of *E. coli* NCM631 harboring the plasmid pIZ227^41^ and either pMPO784, (*thnA4-His*_*6*_), pMPO760, (*thnA3-His*_*6*_) or pMPO785 (*His*_*6*_*-thnY),* were grown in LB medium containing ampicillin and chloramphenicol overnight. They were used to inoculate 1 liter of Terrific Broth medium^29^ supplemented with extra iron and sulfur sources (0.1 mg/ml ferric ammonium citrate, 0.1 mg/ml ferric citrate, 0.1 mg/ml iron sulfate heptahydrate, 0.1 × ferrous sulfate/chelate solution, and 1 mM cysteine), and 4 mg/ml riboflavin for His_6_-ThnY and ThnA4***-***His_6_. Expression of *thnA3-His*_*6*_ or *thnA4-His*_*6*_ was induced by adding 0.5 or 0.05 mM of isopropyl β-D-thiogalactopyranoside respectively, for 15 h at 16 °C. For ThnA4***-***His_6_, it was necessary to add 2 mM FAD during the cell disruption by sonication to obtain a ferredoxin reductase with both, the iron sulfur-cluster and flavin, cofactors bound. Cell extracts were applied to an IMAC column (TALON^TM^ resin (*Clontech*)) according to the manufacturer’s specifications and bound proteins were eluted with an increasing imidazole gradient. Fractions exhibiting the expecting size and with color, indicative that some chromophores must be bound, were pooled, concentrated and applied to a Superdex 75 pg column (10/60). Purified proteins were stored at −80 °C in 50 mM Tris-HCl buffer, pH 7.4 containing 250 mM NaCl, and 10% glycerol. To measure His_6_-ThnY and ThnA4-His_6_ concentrations, the extinction coefficient of each protein (ε_454 nm_ = 21.8 M^−1 ^cm^−1^) and (ε_461 nm_ = 19.5 M^−1 ^cm^−1^) respectively, were calculated using the same procedure described for ThnY-His_6_^9^. ThnA_3_-His_6_ concentration was determined by the Bradford method using bovine serum albumin as the standard. The application of different methods (including absorption spectroscopic methods and Fe quantification by inductively coupled plasma) in the attempt to determinate the protein extinction coefficient, indicated that [2Fe-2S] cluster incorporation was incomplete, with an average of 35% of incorporation. This prevented determination of the extinction coefficient. UV-Vis spectra were recorded either in a Shimadzu UVPC-1603 or in a Cary-100 spectrophotometer.

### Stopped-flow pre-steady-state kinetic measurements

Transient electron transfer reactions among different couples of the Thn proteins, as well as from NAD(P)H to all of them were analyzed (reduction by NAD(P)H was only efficiently observed for ThnA4) by following the absorption spectral evolution in the flavin and iron sulfur regions (400–900 mm) using an Applied Photophysics SX17MV stopped-flow equipped with a photodiode array detector at 15 °C^30^. All samples were prepared in potassium phosphate 50 mM, pH 7.4, NaCl 10 mM, glycerine 5%. Multiple wavelength absorption data were collected and processed using the X-Scan software (*Appl. Phot. Ltd*). Time spectral deconvolution was performed by global analysis and numerical integration methods using Pro-Kineticist (*Appl. Phot. Ltd.*). The collected data were fitted either to single-step, A → B, two-steps, A → B → C, three-steps, A → B → C → D, or four-steps A → B → C → D → E models allowing estimation of the observed conversion rate constants (*k*_A→B_, *k*_B→C,_ *k*_C→D_). These observed rate constants describe the reaction under a particular set of conditions, being not limiting values, and are estimated with errors lower than ±10%^31^. However, values representing processes involving ThnA_3_ must be considered only from the qualitative point of view due to the low proportion of holoprotein exhibited by this protein. Model validity was assessed by lack of systematic deviations from residual plots at different wavelengths, inspection of calculated spectra and consistence among the number of significant singular values with the fit model. *k*_A→B_ rate constants derived from experimental data for the reaction between NAD(P)H, (2.5–150 μM range), and ThnA4_ox_ showed a dependence profile on the nucleotide concentration that fit to the equation describing binding at a single site followed by the electron transfer:


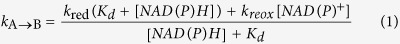


allowing to estimate limiting values at equilibrium for the dissociation (*K*_d_) and the hydride transfer rate constants for the forward and reverse reactions (*k*_red_, *k*_reox_) as previously described^32^,^33^. In general, errors in the estimated values for *K*_d_ and *k*_red_ were lower than ±20% and ±15%, respectively.

### Determination of midpoint reduction potentials for ThnA4, ThnA3 and ThnY

Potentiometric titrations of ThnA4, ThnA3 and ThnY, were attempted at 15 °C by photoreduction under anaerobic conditions. Solutions contained 5 μM ThnA4 or ThnY, or 20 μM ThnA3, as well as 3 mM EDTA and 2 μM 5-deazariboflavin. Measurements were carried out in potassium phosphate 50 mM, pH 7.4, NaCl 10 mM, glycerine 5% for ThnA3 and ThnA4, and in HEPES 0.1 M pH 7.4, guanidine chloride 0.1 M, DTT 1 mM, EDTA 0.1 mM, glycerine 17% for ThnY. As mediators we used benzylviologen (*E*_m_ = −359 mV) and indigo disulphonate (*E*_m_ = −125 mV) for ThnA3; indigo disulphonate, benzylviologen and 1,2-naphtoquinone (*E*_m_ = +143 mV) for ThnY, and benzylviologen, indigo disulphonate and anthraquinone-2-sulphonate (*E*_m_ = −225 mV) for ThnA4. Stepwise reduction of the proteins was achieved by photoreduction and potentials along reduction were determined using a calomel electrode as reference (*E*_m_ = −251.1 mV at 15 °C) and a gold electrode as working one, as previously described^34^. The system was considered equilibrated when the potential of the solution (*E*), measured with a Fluke 177 true-RMS multimeter, remained stable for at least 10 min. UV-vis absorbance spectrum was then recorded. In the case of ThnA4, the extremely slow stabilization of the potential values after each reduction step as well as the subsequent denaturation of the protein upon reduction enforced us to measure potential values and their corresponding absorption spectra after 15 min of each illumination step. This was in detriment of the accuracy in determination of the midpoint potentials for this protein. Experiments were performed in duplicate.

Data were analyzed using the Origin Software (*OriginLab*) based on previously described methods^35^,^36^,^37^,^38^,^39^ using Equation 2 for ThnA3 and Equation 3 for ThnY and ThnA4. These equations, derived by extension to the Nernst equation and the Beer–Lambert Law, respectively describe a one-electron redox process or the sum of a one-electron process and a two-electron redox process;


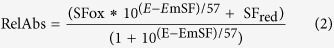



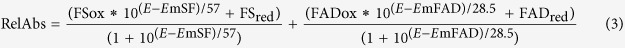


where RelAbs corresponds to the ratio between the sample absorption, at a given wavelength and at a particular stage of the reduction, and the maximal observed for such wavelength; SFe_ox_, SFe_red_, FAD_ox_, and FAD_red_ correspond to the relative contribution to the total RelAbs at each wavelength and stage of reduction of the oxidized and reduced forms of the [2Fe-2S] and FAD redox centers; *E* is the experimentally measured potential at each state of the reduction; and *E*_mSFox/red_ and *E*_mFADox/red_ correspond respectively to the one-electron midpoint potential of the [2Fe-2S] cluster and the two-electron midpoint potential of the flavin cofactor. The complexity of the system, particularly for ThnY and ThnA4 (three-electron titration with probable overlap of midpoint potentials and the presence of several dyes) necessitated the use of a global fitting process. Thus RelAbs at several wavelengths were simultaneously plotted against the redox potential of the solution (mV/SHE) and fit to either Eq. 2 or Eq. 3. Errors in the determined *E*_*m*ox/red_ were estimated to be ±8 mV for ThnA3 and ThnY, but values larger than ±30 mV are expected for ThnA4.

## Additional Information

**How to cite this article**: Ledesma-García, L. *et al*. Redox proteins of hydroxylating bacterial dioxygenases establish a regulatory cascade that prevents gratuitous induction of tetralin biodegradation genes. *Sci. Rep.* **6**, 23848; doi: 10.1038/srep23848 (2016).

## Supplementary Material

Supplementary Information
