# Supplementary material for: Redox proteins of hydroxylating bacterial dioxygenases establish a regulatory cascade that prevents gratuitous induction of tetralin biodegradation genes
Source: Sci Rep. 2016 Mar 31;6:23848. doi: 10.1038/srep23848 (PMC4814904; doi:10.1038/srep23848)
Supplement: Supplementary Information [file srep23848-s1.pdf]

Redox proteins of hydroxylating bacterial dioxygenases establish a regulatory cascade that prevents gratuitous induction of tetralin biodegradation genes.

L. Ledesma-García[1], A. Sánchez-Azqueta[2], M. Medina[2,*], F. Reyes-Ramírez[1,*] and E. Santero[1].

SUPPLEMENTARY INFORMATION

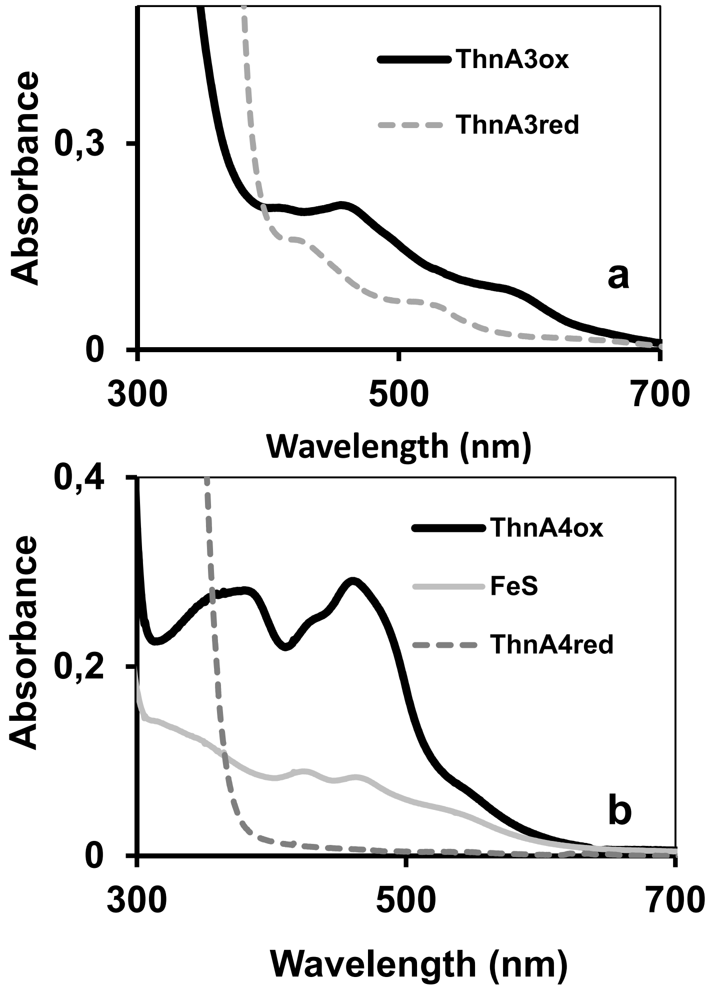

Figure S1. **Visible absorption spectra of ThnA3-His$_6$ (a) and ThnA4-His$_6$ (b) under aerobic conditions.** (a) Oxidized spectrum (black line) of ThnA3-His$_6$ (~45 µM) and the reduced one after addition of sodium dithionite (dashed lines). (b) Spectra of ThnA4-His$_6$ (17 µM) before (grey line, labeled as FeS) and after (black line) reconstitution with FAD. Dashed line, (ThnA4$_{red}$), is the spectrum after the addition of sodium dithionite to the reconstituted protein.

Supplementary Table S1: Plasmids, strains, and primers used in this work

**Strains or plasmids**	**Relevant genotype/phenot**ype	**Reference**
E. coli DH5α	[F^- ϕ80d *lacZ*ΔM15 Δ(*lacZYA–argF)U169 recA1 endA1 hsdR17* ($r_k^- m_k^-$) *supE44 thi-1 gypA relA1*]	40
E. coli NCM631	*hsdS gal* λDE3:*lacI lacUV5:gen1*(T7 RNA-polymerase) Δ*lac* linked to Tn*10*	41
**Plasmids**		
pET23b	Expression vector for purification of proteins by metal affinity chromatography. Ap^r	Novagen
pET14b	Expression vector for purification of proteins by metal affinity chromatography. Ap^r	Novagen
pIZ227	pACYC184 containing *lacI^q* and the T7 lysozyme gene. Cm^r	41
pMPO750	*thnY* into pIZ1016. Gm^r	9
pMPO751	*thnA3A4RY* into pIZ1016. Gm^r	11
pMPO785	pET14b plasmid overexpressing His_6-ThnY. Ap^r	This work
pMPO760	pET23b plasmid overexpressing ThnA3-His_6. Ap^r	This work
pMPO784	pET23b plasmid overexpressing ThnA4-His_6. Ap^r	This work
